# Isolation of the highly pathogenic and zoonotic agent *Burkholderia pseudomallei* from a pet green Iguana in Prague, Czech Republic

**DOI:** 10.1186/s12917-014-0283-7

**Published:** 2014-11-28

**Authors:** Mandy C Elschner, Jan Hnizdo, Ivonne Stamm, Hosny El-Adawy, Katja Mertens, Falk Melzer

**Affiliations:** Friedrich-Loeffler-Institut, Federal Research Institute for Animal Health, Institute of Bacterial Infections and Zoonoses, Naumburger Strasse 96a, 07743 Jena, Germany; Animal Clinic, Bílá Hora, Cistovicka 44, 16300 Prague 6, Czech Republic; Vet Med Labor GmbH, Division of IDEXX Laboratories, Mörikestrasse 28/3, 71636 Ludwigsburg, Germany

**Keywords:** Melioidosis, *Burkholderia pseudomallei*, Zoonoses, Iguana

## Abstract

**Background:**

*Melioidosis caused by Burkholderia (B.) pseudomallei* is an endemic zoonotic disease mainly reported from northern Australia and Southeast Asia. In Europe, cases of human melioidosis have been reported only from patients travelling to endemic regions. Besides humans, *B. pseudomallei* has a very broad host range in domestic and wild animals. There are some reports about importation of *B. pseudomallei*-infected animals from endemic areas into Europe. The present report describes the first case of *B. pseudomallei* infection of a pet iguana in Europe.

**Case presentation:**

In a 5-year-old pet *Iguana iguana* living in a private household in Prague, Czech Republic, *B. pseudomallei* was isolated from pus of an abscess. The isolate VB976100 was identified by Vitek®2, MALDI-TOF mass spectrometry and polymerase chain reaction as *B. pseudomallei*. The molecular typing resulted in multi-locus sequence type 436 hitherto, which has been found only once worldwide in a *B. pseudomallei* strain isolated in the USA and originating from Guatemala. The identification as internal transcribed spacer type G indicates a close relatedness to strains mainly isolated in the Western Hemisphere. These findings support the hypothesis that the iguana became infected in this region or in a breeding facility through contact to other infected animals.

**Conclusions:**

The present case highlights the risk of importation of the highly pathogenic and zoonotic *B. pseudomallei* into non-endemic regions through animal trade. Therefore, veterinarians treating animals from these areas and physicians examining patients owning such animals should include melioidosis in differential diagnosis whenever specific symptoms appear. Furthermore, veterinary authorities responsible for supervision of traders and pet shops should be aware of this risk of zoonotic transmission.

## Background

*Burkholderia (B.) pseudomallei* is the causative agent of melioidosis, an endemic disease mainly reported from northern Australia and Southeast Asia. In this area the disease has emerged as an important cause of morbidity and mortality during the last 25 years [1]. Endemic and sporadic cases of melioidosis are also reported from countries of South America, North America, Oceania, as well as Aruba, Guadeloupe, Guam, Haiti, Martinique, and Puerto Rico. Increasing numbers of cases are reported from Africa [2]. However, in Europe cases of human melioidosis have only reported from patients who had been travelling to endemic regions [3,4]. The gram-negative bacterium belongs to biohazard risk group 3 agents, is listed as biowarfare agent, and has to be processed under biosafety level 3 conditions. The disease is mainly acquired environmentally by percutaneous infection, ingestion, or inhalation. The incubation period of melioidosis can vary between one day and 62 years, depending on the route of infection and infection dose [1]. Clinical studies have shown that pneumonia is the predominant clinical manifestation, followed by skin and soft tissue infections, as well as acute suppurative parotitis, especially in pediatric cases, and prostatitis in males [5]. One predominant risk factor is diabetes mellitus, as shown by studies in Australia and Thailand, where up to 60% of the melioidosis patients are diabetic, mainly type 2 [5].

Besides humans, *B. pseudomallei* has a very broad host range. In domestic animals it is most commonly reported in cattle, goats and swine [6]. However, sporadic cases or small outbreaks have been reported in monkeys, gibbons, orangutans, kangaroos, wallabies, deer, buffaloes, cows, camels, llamas, zebras, koalas, dogs, cats, horses, mules, parrots, rats, hamsters, rabbits, guinea pigs, ground squirrels, seals, dolphins, crocodiles [7]. Very recently, *B. pseudomallei* infections in two pet iguanas in California were reported [8]. In animals, acute and chronical form of melioidosis is seen. Common symptoms in animals include anorexia, pyrexia, coughing, skin dehydration and abscesses [7,9].

## Case presentation

**Figure 1:**
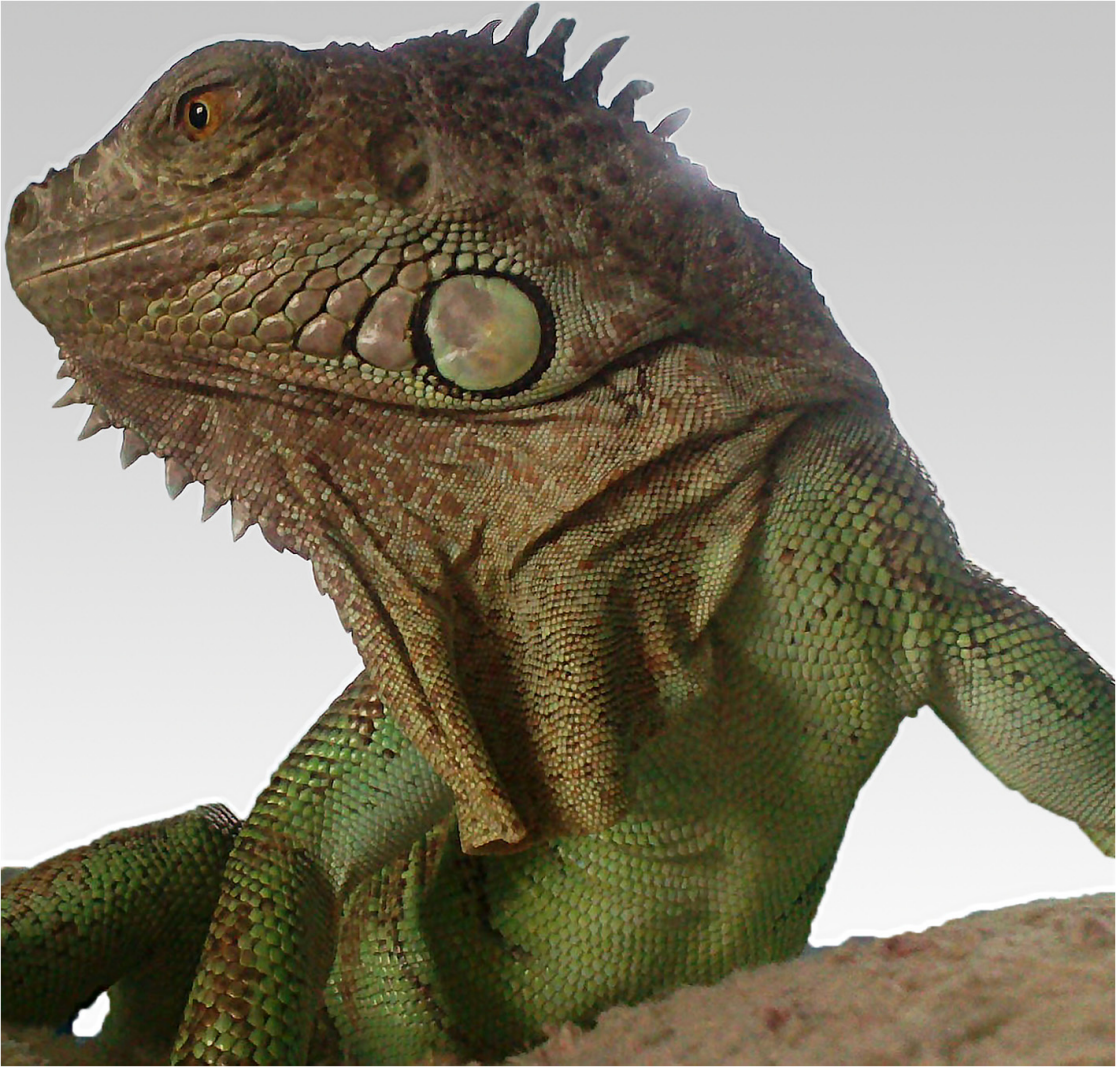
**The infected animal: 5-year-old female green iguana (** ***Iguana iguana*** **).**

The female *Iguana iguana* was purchased by private owners as a young animal from a pet shop in Prague in 2009. It was kept alone in a terrarium, but was also allowed to move freely outside the cage in the flat (Figure 1). The owners had a very close and tender contact to the animal, such as hand feeding and kissing. Usually the iguana was fed with special pellet formulation for iguanas, fresh green salad, vegetables and fruits, and once a month by *Zophobas morio* larvae purchased from different breeders. In 2011, the animal developed an abscess underneath the left eye, which was transected and treated antibiotically without microbiological investigation. In 2013, the animal was admitted to the Animal Clinic, Bila Hora, Prague, Czech Republic, where again an abscess was diagnosed, this time at the root of the tongue. After surgical intervention and antibiotic treatment by marbofloxacine the animal recovered. One year later, in 2014 the owners presented the animal again to the clinic because of a week-long anorexia. The clinical investigation resulted again in an encapsulated abscess located at the root of the tongue. This time the abscess was sampled for microbiological analysis. As soon as the first suspicion of a *B. pseudomallei* infection was announced, the veterinary authority was informed, and it mandated the immediate euthanization and incineration of the animal. One veterinary assistant was bitten by the iguana when he tried to fix the maxilla for sampling of the abscess by the veterinarian. The skin was injured although he wore gloves. Finally, the wound was cured and the assistant was prophylactically treated with amoxicillin-clavulanate for 10 days in a clinic specialized for infectious diseases. Also the owners, not showing any clinical signs, were referred to a physician for medical examination and received the same treatment. Serological investigations were not induced in the contact persons, because the physicians could not find a laboratory providing this analysis. Unfortunately, no information about follow up examinations of the contact persons is available.

### Isolation, identification, and molecular characterisation of *B. pseudomallei*

**Figure 2:**
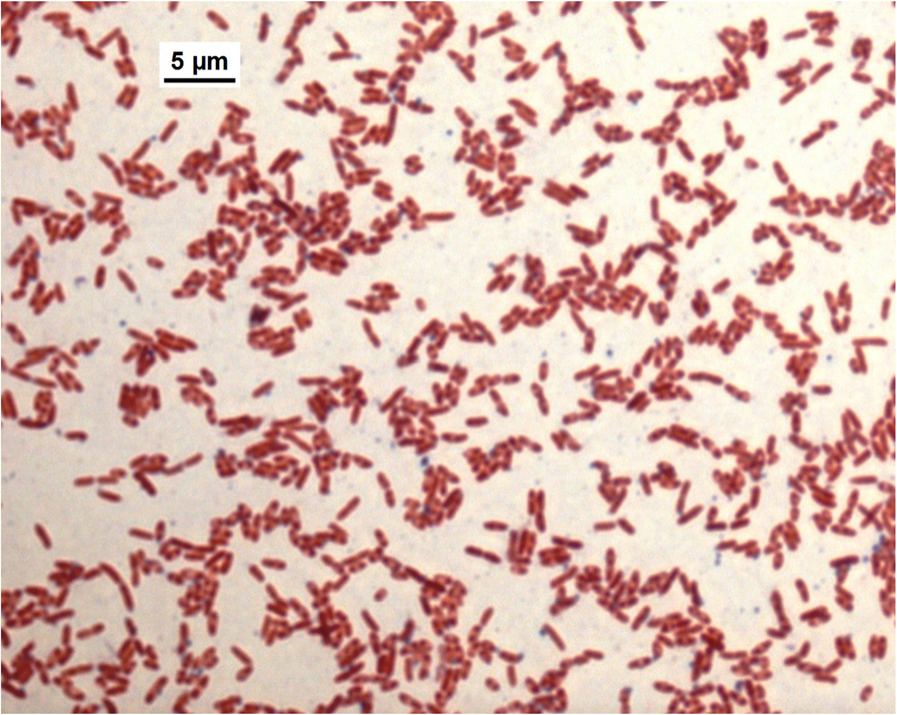
**Gram-stain of** ***B pseudomallei*** **isolate VB976100, phase contrast microscopy with a 100 x oil immersion objective (Microscope Leica DM4000B).**

For microbiological diagnosis, the pus from the abscess was submitted on a swab with Amies transport medium (D&D Laborservice, Henningsdorf, Germany) to the Vet Med Labor GmbH, Ludwigsburg, Germany. The sample was cultured on MacConkey agar (BioMerieux, Nürtingen, Germany), tryptic soy agar with 5% sheep blood, chocolate agar (both BD, Heidelberg, Germany) and incubated at 36°C under atmosphere with 5% CO_2_ for 24 hours, and afterwards for 24 hours in ambient atmosphere. Additionally, brain heart infusion enrichment broth was inoculated and sub-cultured for 24 hours on the same agar media as above. Heavy growth of metallic gleaming navel forming colonies was visible in pure culture after 48-h incubation on all culture media. The isolate VB976100 could also be grown at 42°C, but not at 4°C. It showed a positive Oxidase reaction (VWR, Darmstadt, Germany) and was tested negative for catalase (Sigma-Aldrich Chemie GmbH, Munich, Germany). Short gram-negative rods with bipolar “safety pin” appearance were visible after Gram staining (Figure 2). Identification with Vitek®2 (BioMerieux) resulted in *B. pseudomallei* with 95% probability, corresponding to a very good validity of identification. The same identification result was obtained with MALDI-TOF mass spectrometry (Biotyper Microflex LT, Bruker Daltonics GmbH, Bremen, Germany) with very high score values of >2.600 corresponding to highly probable species identification.

**Figure 3:**
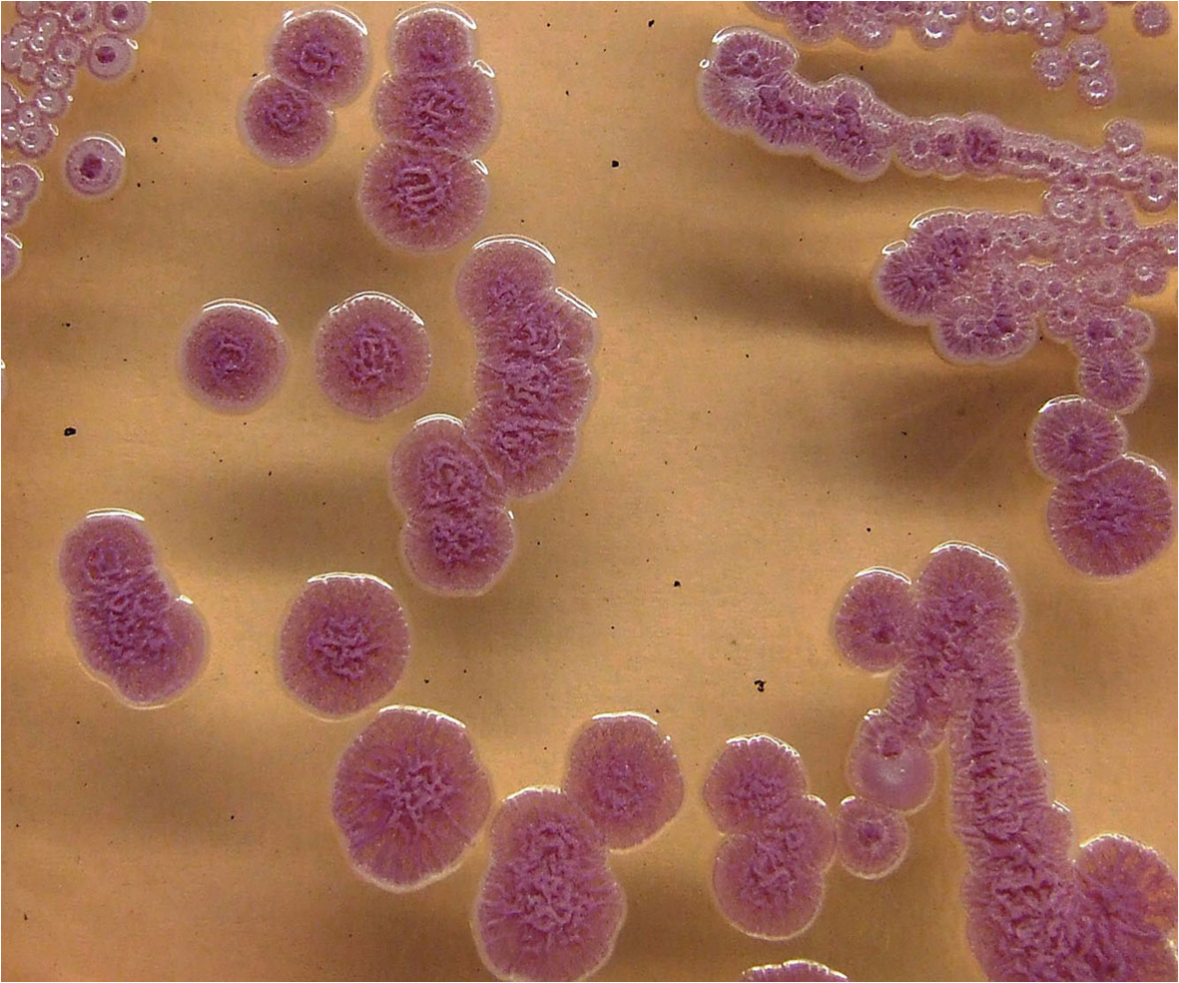
**Colonies of** ***B. pseudomallei*** **isolate VB976100 on Ashdown agar showing the typical pink crinkled colonies after 72 h incubation.**

For confirmation of *B. pseudomallei* and further characterization, the culture was sent to the Federal Research Institute for Animal Health, Jena, Germany. The isolate VB976100 was cultured under biosafety level 3 conditions and the typical shape of colonies was seen on Ashdown-Agar [10] after 72 h (Figure 3).

Molecular identification was performed by real-time PCR detecting *B. mallei/pseudomallei*-specific sequences of the *fliC* gene [11] and *B. pseudomallei*-specific sequences of the *orf2* gene, which belongs to the type III secretion system [12].

Multi-locus sequence typing (MLST), the predominant method for molecular subtyping of *B. pseudomallei* was performed by comparison of 7 housekeeping genes [13]. The isolate was typed as sequence type (ST) 436 by the *B. pseudomallei* database of MLST.Net [14], and data have been deposited there. This ST has been only isolated in California from a patient originating from Guatemala in 2012 [15]. However, for an isolate of unknown origin, MLST alone is not adequate for determination of the geographic origin [15,16]. Therefore, we performed the 16S-32S internal transcribed spacer (ITS) typing [16]. For identification of the ITS type, the obtained sequence data were aligned to known ITS types A, B, C, D, E, and G, available data at GenBank (FJ981703-F981726) using the Software Geneious 6.1.8. (Biomatters Development Team). Our *B. pseudomallei* isolate VB976100 was typed as ITS type G (613 base pairs), and the ITS sequence was annotated and submitted to GenBank under the accession number KP069478.

## Conclusions

Import of *B. pseudomallei* infected animals from endemic areas has been previously reported in sheep, goats and pigs from Aruba [17]. In the 1970s a *B. pseudomallei* outbreak was reported in the zoo of Paris, and the infection was spread to other zoos and equestrian clubs. In the course of this outbreak, termed “l’affaire du jardin des plantes”, two individuals died. Imported horses from Iran or an imported panda from China were assumed as sources of infection [18,19].

*B. pseudomallei* infections in green iguanas were already detected in California, USA, in 2007 and 2012. The bacteria were isolated from the abscess, as well as from the animal housing [8]. In both cases the isolates were typed as ST 518 and ITS type G. The authors suggested that the animals had been infected in Central America, where most imported iguanas in the USA originate from. Furthermore, these cases already highlighted the possible risk of human infections because the infection in the iguanas was not curable in spite of refractory antibiotic treatment. In one of these cases after the incubation period of around 1.5 years an abscess was diagnosed. In the clinical course of the present reported *B. pseudomallei* infection, the animal developed abscesses at the age of 2, 4 and 5 years. However, there was no known history of any kind of trauma, which potentially could have triggered the development of abscesses [8,20]. Unfortunately, only in 2014 bacteriological examination was initiated. It is speculative but possible that after an incubation period of 2 years the first abscess was caused by a *B. pseudomallei* infection. These finding strongly supports the presumption, that in these animals a long incubation period is seen, bearing a risk of long not recognized phase of shedding and herewith infection risk for contact persons.

The iguana was purchased as a young animal in 2009, and unfortunately, it was not possible to determine the origin of this animal*.* However, the ST 436 was hitherto described for one *B. pseudomallei* strain, isolated in USA, originating from Guatemala [15]. On the other hand, the ITS type G of *B. pseudomallei* is a less common type in endemic regions, such as Australia and Southeast Asia. This type seems to be associated with isolates found only in sporadic melioidosis regions like Africa and South America [15,16]. These findings support the hypothesis that the iguana became infected in this region or in a breeding facility through contact to other infected animals originating from this area.

Our report emphasizes the risk of importation of *B. pseudomallei* into non-endemic regions through animal trade. It is possible that for patients presenting characteristic symptoms of melioidosis but with absolutely no history of travelling to endemic regions, the attending physician could misdiagnose or overlook the suspicion of melioidosis. The utility of a post exposure prophylaxis in contact persons should be discussed with the physicians. Recommendations for prophylaxis and treatment are available from Bossi et al. [21].

Personal safety can be improved by wearing eye protection and suitable gloves during sampling and treatment of possibly affected animals. For the serological investigations in non-endemic areas IgG ELISA could be useful to identify chronically infected persons [22]. Since no standardized commercial tests are available only specialized laboratories can perform such investigations.

Although we are unable to prove the source of infection in the present case, veterinarians involved in treatment of animals and competent veterinary authorities responsible for supervision of traders should be aware of this potential risk.

## Abbreviations

B: *Burkholderia*
MLST: Multi-locus sequence typing
ITS: Internal transcribed spacer typing
ST: Sequence type
PCR: Polymerase chain reaction
